# Esophageal Perforation following Accidental Ingestion of a Razor Blade

**DOI:** 10.1155/2022/1974147

**Published:** 2022-03-17

**Authors:** Suraj Shrestha, Ranjan Sapkota, Suraj Bhatta, Sanjeev Kharel, Bibek Man Shrestha, Aakriti Sharma

**Affiliations:** ^1^Maharajgunj Medical Campus, Institute of Medicine, Kathmandu, Nepal; ^2^Department of Cardio-Thoracic and Vascular Surgery, Manmohan Cardio-Thoracic Vascular and Transplant Center, Kathmandu, Nepal

## Abstract

**Background:**

Ingestion of sharp foreign bodies is uncommon and often underreported. It can present with esophageal perforation which is a life-threatening complication requiring prompt diagnosis and management. *Case Presentation.* We report a case of accidental ingestion of a razor blade in a chronic alcoholic who presented with hematemesis after an esophageal perforation, the diagnosis of which was confirmed by radiology.

**Conclusion:**

Early recognition of esophageal perforation is crucial for early intervention. Proper history taking and radiological investigations are a key to reaching a diagnosis.

## 1. Background

Ingestion of foreign bodies (FB) is common in children, while in adults it is commonly seen among those with psychiatric disorders, mental retardation, prisoners, and alcoholics [1]. The ingestion of a sharp foreign body like a razor blade can be catastrophic and may be associated with numerous complications such as perforation, mediastinitis, subcutaneous emphysema, aspiration, mediastinal abscess, bleeding, complete bowel obstruction, and pressure necrosis with subsequent aortic/tracheal fistula formation. This mandates a prompt diagnosis and urgent removal of these objects [2].

Reports of ingestion of sharp objects, more specifically razor blades, are rare in the literature. Herein, we report a case of a chronic alcoholic who accidentally ingested a razor blade and presented with esophageal perforation.

## 2. Case Presentation

A 58-year-old male was referred to our center with a two-day history of acute anterior chest pain radiating to the shoulder, dysphagia, and multiple episodes of hematemesis following probable ingestion of a foreign body under the influence of alcohol. The patient was apparently unaware of the incident and timing of the ingestion. There was no history of fever, loss of consciousness, body discoloration, significant weight loss, dysphagia, and similar events in the past. He had been consuming approximately 1.5 liters of homemade alcohol every day for the last 40 years. There is no history of intake of psychoactive substances and of any documented psychiatric illness.

On examination, the patient was ill-looking, alert, and afebrile with a blood pressure of 110/80 mmHg, pulse rate of 100 beats per minute, respiratory rate of 28 per minute, and SpO_2_ of 82% in room air with right intercostal drain and nasogastric tube in situ draining blood mixed contents. On chest auscultation, air entry was reduced bilaterally without any added sound. The abdomen was soft, and examination of other systems was unremarkable.

**Figure 1:**
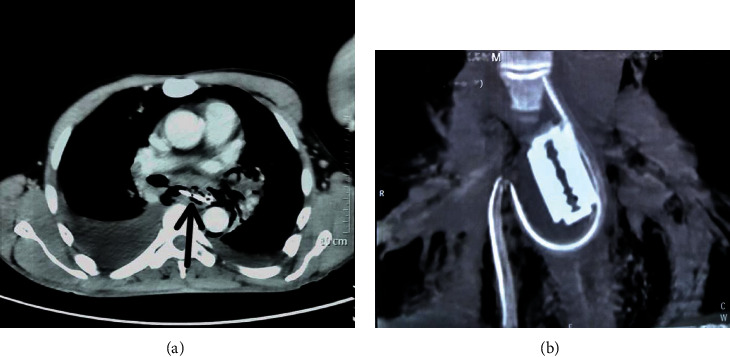
CT chest. (a) Sharp razor blade (arrow) in the mediastinum with pneumomediastinum and moderately large right pleural effusion. (b) Reconstructed image shows the whole blade; apparently, the NG tube traveled through the mediastinum.

Chest X-ray revealed pneumomediastinum, pneumopericardium, and right-sided pleural effusion with a tube in the right chest. Contrast-enhanced computed tomography (CECT) of the chest and abdomen revealed a 5 cm × 2 cm double-edged shaving razor blade in the midesophagus causing a 45 mm perforation at its lateral aspect at T_6-8_ level with extensive pneumomediastinum and surgical emphysema in the neck along with bilateral pleural effusions (Figure 1).

**Figure 2:**
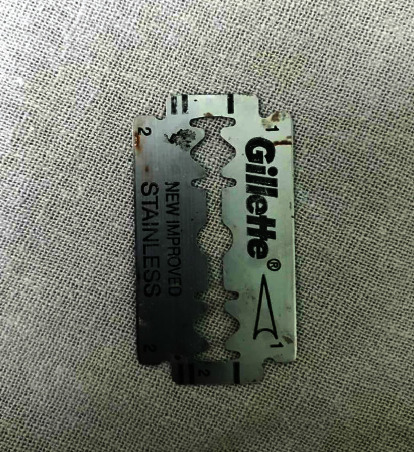
The razor blade after removal.

**Figure 3:**
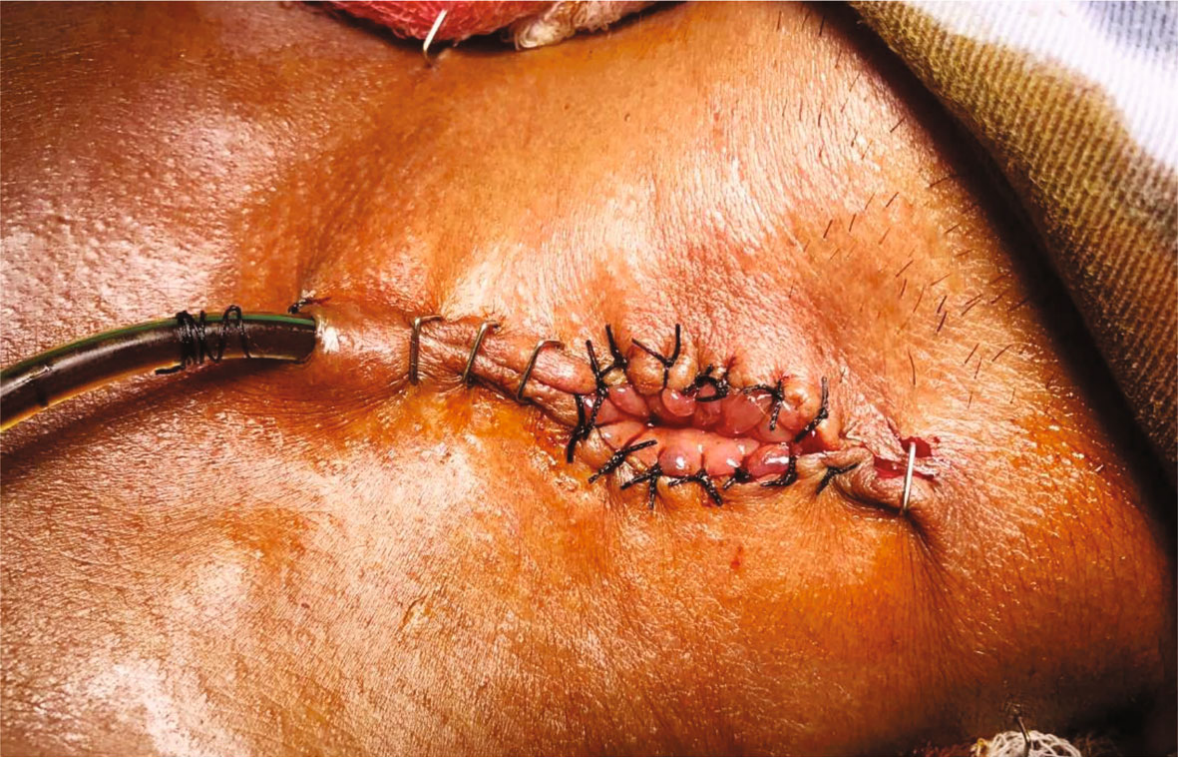
Exteriorized esophagus.

With a diagnosis of razor blade ingestion causing thoracic esophageal perforation, a decision was taken to pursue operative management. The patient underwent a right-sided posterolateral thoracotomy. The blade was removed intact, the pleural cavity was cleared of pus and debris (Figure 2). The mediastinum and the pleural cavity were thoroughly washed, and the chest closed with a drain. The left neck was explored and the esophagus was exteriorized (Figure 3). A feeding jejunostomy (FJ) was done via a small laparotomy. After an ICU stay of 3 days, he recovered well. At discharge, he was self-ambulating, receiving approximately 1500 Kcal diet per day via FJ and off oxygen. A gastric pull-up operation has been planned in about six weeks' time.

## 3. Discussion

Foreign body ingestion is not an uncommon entity. Individuals under the influence of drugs and/or alcohol often present to the emergency after ingesting multiple foreign bodies. The ingestion tends to be spontaneous, and frequently, patients do not remember swallowing the object [3]. Our patient was too unaware of the incident.

One-third of foreign bodies retained in the gastrointestinal tract are present in the esophagus [4]. The duodenal loop, duodenojejunal junction, ileocecal valve, and appendix are other potential areas where it can get lodged due to anatomical narrowing or acute angulations [5]. These ingested sharp objects that are retained in the esophagus carry the risk of perforating the esophagus leading to acute mediastinitis, acute bleeding, or rarely abscess or fistula formation, which may even result in death and are thus considered by far the most dangerous [6, 7].

The common presenting complaint in esophageal perforation is pain in the neck, chest, abdomen, or shoulder, depending upon the location of the perforation. Episodes of forceful or repeated vomiting may precede pain. In about 25% of the patients, pain is followed by vomiting and dyspnea. Dyspnea is more common in thoracic esophageal perforations. The presence of the Mackler's triad, a history of forceful emesis, subxiphoid chest pain, and subcutaneous emphysema often suggests acute esophageal rupture [8]. Our patient had chest pain, dysphagia, hematemesis, and subcutaneous emphysema in the neck.

For those who ingested FB accidentally under the influence of alcohol as in our case, medical evaluation can be difficult as they often cannot provide a reliable history [9]. Because of the aforementioned dreadful complications, patients with suspected esophageal perforation should be regarded as critically ill. Early recognition with timely intervention is crucial for early recovery.

Chest and abdominal X-rays can elicit a radiopaque FB-like metallic material among suspected esophageal FB [10]. However, it was not easily visualized in the X-ray of our patient. As relevant to our case, if a patient is unable to provide a satisfactory history and chest X-ray is inconclusive, other modalities of diagnosis like CT scan and diagnostic endoscopy are generally the preferred modalities. Pleural effusion, pneumomediastinum, subcutaneous emphysema, hydrothorax, and pneumothorax are some other indirect signs of esophageal injury that aid in the diagnosis [11]. Apart from demonstrable perforation, our patient also had a bilateral pleural collection, pneumomediastinum, and subcutaneous emphysema in the neck.

Of all the FB ingested, about 20% need endoscopic extraction and surgical interventions are required in only less than 1% of presentations while the remaining pass through the gastrointestinal tract uneventfully [12]. However, with the ingestion of sharp FBs, the need for surgical intervention is as high as 35% as their complications are high and if retained in the esophagus can be life-threatening [4, 13]. Thus, extraction of the foreign body as soon as the diagnosis is made is mandatory [4]. As esophageal perforation is a thoracic emergency, surgical treatment is the rule. Hence, aggressive operative intervention remains the mainstay for the treatment [14, 15]. Further, extraction of the foreign body, enteric without oral feeding, antibiotics, and drainage of collections constitute the treatment strategy [16]. The exclusion and diversion of the esophagus promote healing in addition to minimizing the risk of further contamination and infection [17]. Our patient was kept nil orally after suspected esophageal perforation, underwent emergency thoracotomy with FB removal along with esophageal diversion and insertion of an FJ.

In addition to the medical and surgical treatment, psychiatric evaluations are necessary in cases of intentional and/or repeat foreign body ingestion [18]. Our patient was evaluated for alcohol dependence syndrome while no other psychiatric illness was found.

## 4. Conclusion

Ingestion of sharp objects causing esophageal perforation is a life-threatening condition. Prompt diagnosis is imperative for favorable results.

## Data Availability

All the necessary information are provided within the case report.

## Abbreviations

CECT: Contrast-enhanced computed tomography
FB: Foreign body
FJ: Feeding jejunostomy
ICU: Intensive care unit.

## Supplementary Materials

Supplementary Materials CARE-checklist.
