# Supplementary material for: Esophageal Perforation following Accidental Ingestion of a Razor Blade
Source: Case Rep Surg. 2022 Mar 17;2022:1974147. doi: 10.1155/2022/1974147 (PMC8947916; doi:10.1155/2022/1974147)
Supplement: Supplementary Materials — CARE-checklist. [file 1974147.f1.docx]

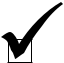

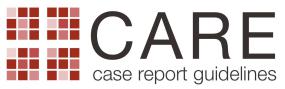
**CARE Checklist of information to include when writing a case report**

**
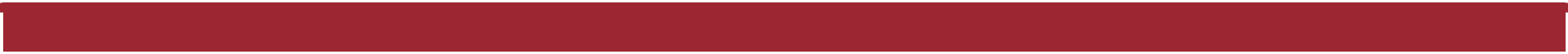

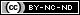
**


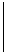
 **Topic**

**Title**

**Key Words**

**Abstract**

**(no references)**

**Introduction Patient Information**

**Clinical Findings**

**Timeline**

**Diagnostic**

**Assessment**

**Therapeutic**

**Intervention**

**Follow-up and Outcomes**

**Discussion**

**Patient Perspective Informed Consent**

| **Item** | **Checklist item description** |  |
| --- | --- | --- |
| **1** | The diagnosis or intervention of primary focus followed by the words “case report” . . . . . . . . . . . . . . . . . . . . . . . . | . . |
| **2** | 2 to 5 key words that identify diagnoses or interventions in this case report, including "case report" . | . . |
| **3a** | Introduction: What is unique about this case and what does it add to the scientific literature? . . . . . . . . . . . . . . | . . |
| **3b** | Main symptoms and/or important clinical findings . . . . . . . . . . . . . . . . . . . . . . . . . . . . . . . . . . . . . . . . . . . . . . . . . . . . . . . | |
| **3c** | The main diagnoses, therapeutic interventions, and outcomes . . . . . . . . . . . . . . . . . . . . . . . . . . . . . . . . . . . . . . . . . . . | |
| **3d** | Conclusion—What is the main “take-away” lesson(s) from this case? . . . . . . . . . . . . . . . . . . . . . . . . . . . . . . . . . . . . . | |
| **4** | One or two paragraphs summarizing why this case is unique (**may include** **references**) . . . . . . . . . . | . . |
| **5a** | De-identified patient specific information. . . . . . . . . . . . . . . . . . . . . . . . . . . . . . . . . . . . . . . . . . . . . . . . . . . . | |
| **5b** | Primary concerns and symptoms of the patient. . . . . . . . . . . . . . . . . . . . . . . . . . . . . . . . . . . . . . . . . . . . . . . . . . . . . | |
| **5c** | Medical, family, and psycho-social history including relevant genetic information . . . . . . . . . . . . . . . | . . |
| **5d** | Relevant past interventions with outcomes . . . . . . . . . . . . . . . . . . . . . . . . . . . . . . . . . . . . . . . . . . . . . . . . . . . . . . | . . |
| **6** | Describe significant physical examination (PE) and important clinical findings. . . . . . . . . . . . . . . . . . . . . | . . |
| **7** | Historical and current information from this episode of care organized as a timeline . . . . . . . . . . . . . . . | |
| **8a** | Diagnostic testing (such as PE, laboratory testing, imaging, surveys). . . . . . . . . . . . . . . . . . . . . . . . . . . . . | . . |
| **8b** | Diagnostic challenges (such as access to testing, financial, or cultural) . . . . . . . . . . . . . . . . . . . . . . . . . . . . . | |
| **8c** | Diagnosis (including other diagnoses considered) . . . . . . . . . . . . . . . . . . . . . . . . . . . . . . . . . . . . . . . . . . . . . . . | . . |
| **8d** | Prognosis (such as staging in oncology) where applicable . . . . . . . . . . . . . . . . . . . . . . . . . . . . . . . . . . . . . . . . . | |
| **9a** | Types of therapeutic intervention (such as pharmacologic, surgical, preventive, self-care) . . . . . . . . . . . . . . . . . . | . . |
| **9b** | Administration of therapeutic intervention (such as dosage, strength, duration) . . . . . . . . . . . . . . . . . . . . . . . . . . . . . | |
| **9c** | Changes in therapeutic intervention (with rationale) . . . . . . . . . . . . . . . . . . . . . . . . . . . . . . . . . . . . . . . . . . . . . . . . . . . . | |
| **10a** | Clinician and patient-assessed outcomes (if available) . . . . . . . . . . . . . . . . . . . . . . .. . . . . . . . . . . . . . . . . . . . . . . . . . | . . |
| **10b** | Important follow-up diagnostic and other test results . . . . . . . . . . . . . . . . . . . . . . . . . . . . . . . . . . . . . . . . . . . . . . . . . . | . . |
| **10c** | Intervention adherence and tolerability (How was this assessed?) . . . . . . . . . . . . . . . . . . . . . . . . . . . . . . . . . . . . . . . | . . |
| **10d** | Adverse and unanticipated events . . . . . . . . . . . . . . . . . . . . . . . . . . . . . . . . . . . . . . . . . . . . . . . . . . . . . . . . . . . . . . . . . | . . |
| **11a** | A scientific discussion of the strengths AND limitations associated with this case report . . . . . . . . . . . . . . . . . . . . . | . . |
| **11b** | Discussion of the relevant medical literature **with references**. . . . . . . . . . . . . . . . . . . . . . . . . . . . . . . . . . . . . . . . | . . |
| **11c** | The scientific rationale for any conclusions (including assessment of possible causes) . . . . . . . . . . . . . . . . . . . . . . . . | |
| **11d** | The primary “take-away” lessons of this case report (without references) in a one paragraph conclusion . . . . . | . . |
| **12** | The patient should share their perspective in one to two paragraphs on the treatment(s) they received . . . . . . . . | |
| **13** | Did the patient give informed consent? Please provide if requested . . . . . . . . . . . . Yes. . . . . . . . . . . . . . . . . . . . . . . . | . . |

**Reported on Line**

**
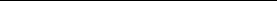

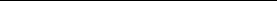

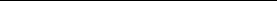

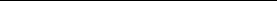

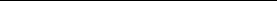

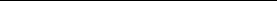

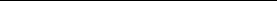

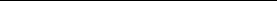

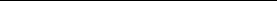

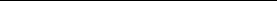

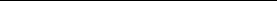

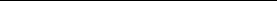

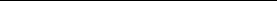

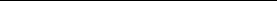

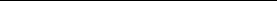

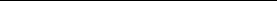

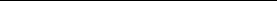

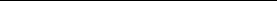

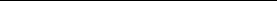

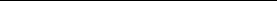

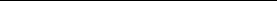

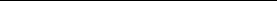

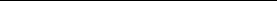

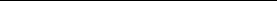

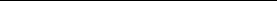

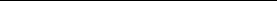

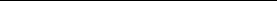

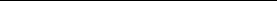

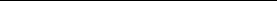
**

1-2

30

16-19

22

23

26-28

33-40

45

46-49

50-52

51-52

53-57

45,53

58-62

63-64

63

NA

64-68

69-70

69-70

NA

70-71

69

68

74-114

78-89,103-113

116-118

119-120

NA

**Yes
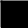

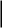
 No**

**
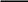

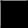
**
